# Metagenomic profiling reveals that divergent fire-adaptive strategies drive distinct fungal succession in pine litter decomposition

**DOI:** 10.3897/imafungus.17.201414

**Published:** 2026-10-06

**Authors:** Yan-Han Fang, Hsin-Ni Liu, Yen-Ju Chen, Tsai-Wen Hsu, Ching-An Chiu, Po-Neng Chiang, Chao-Li Huang

**Affiliations:** 1 Graduate Program in Translational Agricultural Sciences, National Cheng Kung University and Academia Sinica, Tainan 701, Taiwan Graduate Program in Translational Agricultural Sciences, National Cheng Kung University and Academia Sinica Tainan Taiwan; 2 Institute of Tropical Plant Sciences and Microbiology, National Cheng Kung University, Tainan 701, Taiwan Institute of Tropical Plant Sciences and Microbiology, National Cheng Kung University Tainan Taiwan; 3 Taiwan Biodiversity Research Institute, Nantou 552, Taiwan Taiwan Biodiversity Research Institute Nantou Taiwan; 4 Department of Forestry, National Chung Hsing University, Taichung 402, Taiwan Department of Forestry, National Chung Hsing University Taichung Taiwan; 5 Experimental Forest, National Taiwan University, Nantou, Taiwan Experimental Forest, National Taiwan University Nantou Taiwan

**Keywords:** Fire adaptation, lignin-degrading fungi, *
Pinus
*, plant litter, shotgun metagenomics

## Abstract

Decomposing litter is a critical driver of fuel bed formation and forest fire regimes, yet how divergent plant fire-adaptive strategies influence litter chemistry and subsequent fungal decomposition dynamics remains poorly understood. Here, we conducted a 6-month reciprocal litterbag experiment by incubating litter from two contrasting pine species—the fire-tolerant *Pinus taiwanensis* and the fire-avoiding *P. morrisonicola*—in both their own native habitat and that of the other species, coupled with genome-resolved metagenomics to track fungal succession and lignocellulolytic functional profiles. We found that the fire-avoiding *P. morrisonicola* exhibited significantly accelerated decomposition, driven by inherently lower initial C:N ratio and less recalcitrant litter, whereas the fire-tolerant *P. taiwanensis* exhibited slower decomposition rates. Metagenomic profiling revealed a distinct functional succession underlying this process. Early decomposition was dominated by *Ascomycota* (e.g., *Leotiomycetes*), which rapidly colonized the litter to initiate biopolymer breakdown. As easily decomposable substrates depleted, a functional shift occurred. Specifically, the highly recalcitrant *P. taiwanensis* litter selectively recruited *Basidiomycota* (e.g., *Agaricales* and *Trechisporales*) equipped with diverse lignin-modifying enzymes, reflected by a late-stage temporal surge in ligninolytic gene abundances. Ultimately, these findings demonstrate that plant fire-adaptive strategies act as a strong filter, shaping litter chemistry to drive specific taxonomic and functional fungal succession. This study provides a mechanistic understanding of the eco-evolutionary linkage between plant traits, microbiome assembly, and ecosystem-level fire dynamics.

## Introduction

Fire has served as a significant evolutionary force in many regions globally, shaping the traits of species adapted to specific fire regimes (Schwilk and Ackerly 2001; Keeley et al. 2011). Within the genus *Pinus*, the two subgenera, *Strobus* and *Pinus*, exhibit distinct evolutionary responses to varying fire regimes. In Taiwan, two dominant *Pinus* species exemplify these divergent strategies: *P. morrisonicola* belongs to the subgenus *Strobus*, while the endemic *P. taiwanensis* belongs to the subgenus *Pinus*. The subgenus *Strobus* is predominantly found in low-nutrient or extreme environments, where low productivity limits the frequency of fire (He et al. 2012; Pausas 2015); as a result, species like *P. morrisonicola* typically lack fire-adaptive traits and act as fire-avoiding species. In contrast, species within the subgenus *Pinus* display two primary fire-adaptive syndromes: fire-resistant and fire-embracing, each associated with specific fire regimes—surface fire and crown fire, respectively (Keeley 2012). Fire-tolerators, a group within the fire-resistant syndrome, survive surface fire by achieving sufficient height and self-pruning to prevent flames from reaching the canopy. Their thick bark further enhances survival by protecting stems from heat damage (Pausas 2015). *P. taiwanensis* is a representative species of this group. Conversely, fire-embracers resprout or regenerate from seeds released by serotinous cones after crown fire, relying on recruitment rather than resistance to fire (Keeley 2012). Global fire regimes are shaped by climates and vegetation, with fire frequency increasing under drought conditions in high-productivity regions (drought-driven fire regimes) and rising fuel loads rendering low-productivity regions more susceptible to wildfire (fuel-limited fire regimes) (Bond and Midgley 2012; Pausas and Ribeiro 2013; Kelley et al. 2019). In terms of ecosystem carbon cycling, the balance between fire and decomposition influences carbon release patterns, steering the system toward either a fire-driven or a decomposition-driven carbon turnover. These dynamics shape the plant community through a feedback loop that incorporates plant traits related to decomposability and flammability (Cornelissen et al. 2017). Leaves with slower decomposition rates often have a higher leaf mass per unit area (LMA) and lower nitrogen concentration, resulting in a slower carbon return but potentially increasing fire risk as litter accumulates on the forest floor (Cornwell et al. 2008). There is a notable correlation between fire-promoting and fire-surviving traits within fire-adaptive syndromes (Schwilk and Ackerly 2001). This relationship may be influenced by the composition of pine needles, where needle length is critical in determining fuel bed flammability (Cornwell et al. 2015). Fire-avoiders typically have short, densely packed needles, effectively suppressing fire, while fire-tolerators possess long needles that create a low-density fuel bed, thereby increasing flammability (Keeley 2012; Cornwell et al. 2015).

Extensive research has demonstrated that litter chemistry is a primary factor in determining decomposition rates and influencing their limiting values. Berg and McClaugherty (2020) proposed a three-stage model that describes the chemical changes in litter and the factors regulating decomposition rates, based on studies of Scots pine. These stages are the carbohydrate-dominated early stage, the lignin-dominated late stage, and the limit-value (humus-near or final) stage. During the early stage of decomposition, numerous studies have found that decomposition rates are positively correlated with concentrations of N, Mg, P, K, Mn, and Ca, while they are negatively correlated with lignin content, tannin levels, and the C:N ratio (Cornelissen 1996; Berg et al. 2000; Hoorens et al. 2003; Zhang et al. 2008; Makkonen et al. 2012; Lin et al. 2019). During the decomposition process, nitrogen is transferred from surrounding sources and immobilized by microorganisms after the leaching phase, leading to an increase in nitrogen concentration and a reduction in the C:N ratio. This continues until a critical C:N ratio is reached, after which nitrogen starts to be mineralized and released (Fog 1988; Fioretto et al. 2005; Xiong et al. 2014; Purahong et al. 2016; Baskaran et al. 2019; Berg and McClaugherty 2020; Hoeber et al. 2020). Although litter chemistry is a primary driver of decomposition rates and influences the selection of decomposer microbes, the microbiome—shaped by environmental conditions and historical legacies—can exert independent control over decomposition rates, especially among closely related litter species (Allison et al. 2013; Cleveland et al. 2014; Bradford et al. 2016).

*Fungi* are widely studied for their multiple roles in the decomposition of litter and wood, often surpassing bacteria in their contribution to these processes (Osono 2007; van der Wal et al. 2013; Hu et al. 2021). *Fungi* involved in wood decomposition are generally categorized into three types, white-rot, brown-rot, and soft-rot fungi, based on their degrading capacities and the decay patterns. Most white-rot and brown-rot fungi belong to the *Basidiomycota*, whereas soft-rot fungi are primarily classified under *Ascomycota* (Lundell et al. 2014). Fungal species exhibit diverse mechanisms for cellulose degradation, but not all fungi are capable of fully degrading lignin (Berg and McClaugherty 2020). White-rot fungi possess a broad range of degradative abilities, including the complete mineralization of lignin (Berg 2014; Berg and McClaugherty 2020; Janusz et al. 2017). In contrast, brown-rot fungi lack heme-containing peroxidases such as manganese peroxidase (MnP), which are typically found in white-rot fungi. Instead, brown-rot fungi modify lignin by non-enzymatic, low-molecular-weight compounds, resulting in a more reactive lignin with reduced methoxyl groups and increased phenolic hydroxyl groups (Janusz et al. 2017; Berg and McClaugherty 2020). Soft-rot fungi are the least effective at lignin degradation, as they can only partially modify lignin by breaking down the middle lamella of the cell wall (Lundell et al. 2014; Berg and McClaugherty 2020). Lignin degradation involves two groups of enzymes: lignin-modifying enzymes (LMEs, including copper oxidases [CuO], lignin peroxidases [LiP], extradiol ring-cleavage dioxygenase [LigB], and dye-decolorizing peroxidases [DyP]) and lignin-degrading auxiliary enzymes (LDAs, including glucose dehydrogenases [GDH], aromatic peroxygenases [APO], aryl-alcohol oxidases [AAO], glyoxal oxidases [GLOX], and chloroperoxidases [CPO]). LMEs act directly to depolymerize lignin, functioning either independently or synergistically. Conversely, while LDAs cannot degrade lignin alone, they are essential for driving the complete degradation process (Leonowicz et al. 2001; da Silva Coelho-Moreira et al. 2013; Janusz et al. 2017). Ascomycetes tend to dominate in the early stages of decomposition due to their ability to soften and loosen plant tissues, while the more functionally diverse basidiomycetes thrive in the later stages (Voříšková and Baldrian 2013; Purahong et al. 2016; Tan et al. 2020). However, the relationships between decomposition rates and the community composition of lignin decomposers remain under investigation (de Gonzalo et al. 2016).

This study aimed to investigate the effects of needle litter species and plant community on decomposition rates and the composition of litter-decomposing fungal communities. Litterbag experiments were carried out in the *P. taiwanensis* and *P. morrisonicola* forest stands in Taiwan using a fully crossed design with litter from both species. Considering the divergent life strategies and fire-adaptive traits of *P. taiwanensis* and *P. morrisonicola*, we addressed the following questions: (1) Does the fire-avoiding strategy of *P. morrisonicola* inherently lead to accelerated litter decomposition compared to the fire-tolerant strategy of *P. taiwanensis*? (2) Do the forest stand conditions, including local climate factors (e.g., humidity and precipitation) and the stand-associated mycobiomes, act as a secondary driver of litter decomposition by sustaining fungal functions? (3) How do fungal communities temporally partition their cellulolytic and ligninolytic capabilities to accommodate litter with contrasting fire tolerances? Correspondingly, we tested three hypotheses: First, we hypothesized that the fire-avoiding litter of *P. morrisonicola* inherently decomposes faster because its lower structural recalcitrance selects for a more efficient pioneer fungal community. Second, we expected the forest stands to act as a secondary driver by modulating local fungal recruitment. Specifically, drawing upon the home-field advantage (HFA) phenomenon (Ayres et al. 2009; Veen et al. 2019), we inferred that the fire-avoiding species would exhibit an HFA, whereby litter tends to decompose faster in its native environment than in a non-native environment. The rationale for this inference is that fire-tolerant ecosystems inherently promote fire recurrence, which drives frequent soil fungal turnover and limits the long-term establishment of specialized late-stage decomposers natively. Consequently, this recalcitrant litter would decompose more rapidly when exposed to the less-disturbed microbiome outside its native environment. Finally, we hypothesized that fungal communities undergo distinct temporal successions, characterized by the dynamical partitioning of early-stage cellulolytic and late-stage ligninolytic capabilities, to specifically accommodate the distinct decomposition demands of each litter type.

## Materials and methods

### Site description

The study site is located at the DuJuan Ridge in Huisun Experimental Forest Station, National Chung Hsing University (Renai Township, Nantou County, Taiwan). The mean annual precipitation is 2,671 mm (Water Resources Agency, Ministry of Economic Affairs, Taiwan), and the mean annual temperature is 21.0 °C (Huang et al. 2017). Two plots were established: DJM (24°05'29.6"N, 121°02'10.4"E, 755 m elevation, mixed forest of broadleaved trees and *P. morrisonicola*, a fire-avoiding species) and DJT (24°05'39.5"N, 121°02'01.6"E, around 830 m elevation, dominated by *P. taiwanensis*, a fire-tolerant species). These two plots are located on the same hill, separated by approximately 0.4 km. Although they share a continuous landscape with no major geographical barriers between them, they form distinct, spatially segregated forest stands. Historically, this ridge represents a fire-prone ecosystem; prior to our study, the last documented fire event affecting the area occurred on February 28, 1988 (Chen et al. 1989). While each plot was exclusively dominated by its representative living pine species, leaf litters from both pine species were reciprocally incubated at both the DJM and DJT sites. Underscoring the active fire regime of the region, our field experiment was ultimately forced to terminate prematurely due to a severe wildfire that swept through both plots on May 15, 2021. The fire lasted for two weeks, after which no litterbags remained. During the recording period (August of 2020 to February of 2021), the DJT plot had slightly higher daily mean temperature (22.1 ± 3.2 °C) with greater fluctuations in mean maximum (30.0 ± 3.2 °C) and mean minimum (18.0 ± 3.6 °C) compared to DJM (mean: 21.4 ± 3.1 °C; mean max: 26.6 ± 3.0 °C; mean min: 18.0 ± 3.5 °C) (Suppl. material 1: fig. S1). Relative humidity (RH) followed an opposing fluctuation pattern to temperature; DJT exhibited lower daily mean RH (72.3 ± 10.7%) with mean maximum (87.6 ± 10.0%) and mean minimum (49.3 ± 13.2%), whereas DJM had higher values (mean: 75.9 ± 11.4%; mean max: 88.2 ± 10.4%; mean min: 58.4 ± 14.1%; Suppl. material 1: fig. S2). Data gaps led to the exclusion of some recording days, considering data availability from both sites for calculations.

### Litterbag experiment

A reciprocal litterbag experiment was conducted using two native pine species to investigate decomposition dynamics (Suppl. material 1: fig. S3). Fresh fallen needles of *P. taiwanensis* and *P. morrisonicola* were collected on August 4, 2020. The needles were initially dried at 70 °C to a constant mass (Cotrufo et al. 2010; Wang et al. 2025), and 10 g of each litter type was placed into individual litterbags. To ensure a robust spatial distribution, we established five subplots (serving as independent biological replicates) within each of the DJM and DJT plots. The subplots were established along a trail crossing the main plot and spaced at least 5 m apart to reduce the spatial dependence (autocorrelation) of the local mycobiome. Additionally, to minimize potential trail-related disturbances, all subplots were established at least 2 m away from the trail edge. Within each subplot, we deployed 12 litterbags (six containing *P. morrisonicola* litter and six containing *P. taiwanensis* litter) beneath the pine canopies, resulting in 60 litterbags per plot. The original experimental design spanned two years, with planned retrievals at 1, 3, 6, 12, 18, and 24 months. However, due to the aforementioned severe wildfire, the experiment was terminated prematurely. Consequently, all findings are based on the successfully retrieved samples from the first three time points (one, three, and six months; hereafter Harvest 1, 2, and 3). After retrieval, the samples were transported to the laboratory under refrigerated conditions. Upon arrival, 1 g (fresh weight) of the decomposing litter from each bag was weighed and immediately frozen at -20 °C for mycobiome analysis. The remaining material was dried at 70 °C to a constant mass to determine litter mass loss and chemical properties. Soil samples were collected from the surface layer (0–5 cm) immediately around or beneath the litterbags within each subplot in August 2020 and February 2021.

### Litter decomposition and physicochemical properties

To account for the 1.0 g of fresh litter removed for DNA extraction prior to drying, the dry mass equivalent of this 1.0-g fresh subsample was estimated based on the moisture content of the remaining bulk litter. This estimated value was then added to the final measured dry mass to obtain the total dry mass of each litterbag. The decomposition constant was calculated using a single exponential model as described by Olson (1963). The carbon (C), hydrogen (H), nitrogen (N) and sulfur (S) contents in needle litter were determined by combustion method using an elemental analyzer (vario EL cube, Elementar, Italy). The CHNS content was measured for two samples prior to decomposition (representing the two litter species) and after decomposition (2 litter species × 5 replicates × 2 plots × 3 harvests). Changes in chemical shifts in carbon bonds during decomposition were examined using a ^13^C CPMAS NMR spectrometer (Bruker Avance III 400, Bruker, Billerica, MA, USA). The NMR spectra were divided into seven regions representing different carbon types, following Berg and McClaugherty (2020): 0–50 ppm (alkyl-C), 50–60 ppm (methoxyl-C), 60–93 ppm (O-alkyl-C), 93–112 ppm (di-O-alkyl-C), 112–140 ppm (aromatic-C), 140–165 ppm (phenolic-C), and 165–190 ppm (carboxyl-C). Data were quantified by the area under the curve (AUC), calculated using the linear trapezoidal method. Soil samples (2 plots × 5 subplots) were analyzed for CHNS contents, pH, electrical conductivity (EC), and ion concentrations (F^–^, Cl^–^, NO_2_^–^, H_2_PO_4_^–^, Br^–^, NO_3_^–^, SO_4_^2–^, Na^+^, NH_4_^+^, K^+^, Mg^2+^, Ca^2+^) using ion chromatography. Soil water content (SWC) was determined gravimetrically by measuring the weight loss of fresh soil samples (sieved through a 2-mm mesh) after oven-drying at 105 °C to a constant weight.

### Nucleic acid extraction, shotgun metagenomics, and functional annotation

Metagenomic DNA was extracted from decomposing needle litter using the CTAB method (Doyle, 1991), with approximately 160 mg of litter per sample. Litterbag DNA samples from three subplots per plot were analyzed, resulting in a total of 36 samples across three time points and six pairs of litterbags per harvest. Sequencing was performed using 150-bp paired-end reads on the Illumina NovaSeq platform (Illumina, San Diego, CA, USA).

Sample reads were assembled using Megahit v.1.1.3 (Li et al. 2015) after the removal of adaptor sequences by cutadapt v1.10 (Martin 2011). The contigs shorter than 2k were excluded from the following analyses. Reads were mapped back to these contigs for calculating contig abundances via BWA v0.7.12 (Li and Durbin 2009). The mapped results were converted to coverage using SAMtools v0.1.19 (Li and Durbin 2009) and BEDTools2 v2.27.1 (Quinlan and Hall 2010).

To reconstruct fungal metagenome-assembled genomes (MAGs), contigs were binned using CONCOCT v1.1.0 (Alneberg et al. 2014). We specifically selected CONCOCT because its purely algorithmic clustering approach—driven by nucleotide composition and coverage—is highly suitable for eukaryotic genomes, avoiding the biases of other binning tools that are predominantly optimized for prokaryotes. For the resulting fungal MAGs, protein-coding genes were predicted using AUGUSTUS v3.4.0 (Stanke et al. 2008). To optimize gene prediction, we first evaluated all available fungal pre-trained models in AUGUSTUS in a representative subset of our MAGs. This preliminary screening revealed that the best-performing model for any tested MAG invariably belonged to a set of five models (“*aspergillus_oryzae*”, “*botrytis_cinerea*”, “*fusarium_graminearum*”, “*laccaria_bicolor*”, and “*magnaporthe_grisea*”). Subsequently, for each MAG, the specific model—among these five candidates—that yielded the highest completeness score, assessed using BUSCO v4.1.4 (Manni et al. 2021) against the fungi_odb10 lineage dataset, was selected as the standard for final gene prediction. The taxonomic annotation of MAGs was assigned by aligning predicted genes with the NCBI nr database using DIAMOND v2.1.6 (Buchfink et al. 2021) (Suppl. material 1: table S1). Protein functions were annotated using the Pfam database through HMMSCAN (Eddy 1996) and via KEGG prediction by GhostKOALA (Database: Eukaryotes+Prokaryotes+Viruses) (Kanehisa et al. 2016). Lignocellulolytic enzyme information was sourced from the CAZy database domain names (Drula et al. 2022).

### Community and statistical analyses

In R version 4.1.2, fungal MAGs identified by BUSCO were analyzed. Data was normalized using the TMM method (Robinson and Oshlack 2010) through ‘edgeR’ package and community analyses were performed using the ‘vegan’ package (Oksanen et al. 2012). For enzymatic analyses, only MAGs with BUSCO scores >30% were included (Suppl. material 1: table S2). Given the inherent challenges of reconstructing complex eukaryotic genomes from short-read environmental metagenomes, this threshold was strategically selected to balance functional robustness with ecological representation. Assuming an average fungal genome encodes approximately 10,000 protein-coding genes, a >30% completeness threshold ensures the recovery of at least 3,000 genes per MAG, providing a substantial and reliable functional repertoire for profiling lignocellulolytic enzymes. Furthermore, applying a conventional >50% threshold would have excessively constrained our dataset (yielding only 94 MAGs), whereas our >30% threshold retained 139 MAGs for downstream analyses (Suppl. material 1: table S2). To rigorously counteract any quantitative biases caused by partial genome assemblies, all functional gene copy numbers were mathematically normalized by the respective genome completeness of each MAG. We focused on major enzymes of GH5, GH8, GH9, GH1 and GH3, for cellulolytic (Lundell et al. 2014; Wang et al. 2020), and nine ligninolytic enzymes including AAO, APO, CPO, DyP, GLOX, GDH, LiP, laccases (CuO), and LigB (de Gonzalo et al. 2016; Janusz et al. 2017). MAGs carrying MnP or LiP, along with at least two of the other three LMEs, were defined as potential white-rot fungi. As lignin-modifying peroxidases (LiP, MnP, and VP) could not be definitively distinguished, we compared Pfam and KEGG database annotations and incorporated phylogenetic analysis to roughly categorize these LMP-carrying genes into two groups: the MnP clade and the LiP_VP clade (Suppl. material 1: fig. S4). MnP was identified based on KEGG K20205 (mpn), whereas LiP_VP was identified using Pfam annotations for LiP, with sequences corresponding to KEGG entries K23525 (LPO) and K20205 (mpn) excluded. Non-metric multidimensional scaling (NMDS) was employed for compositional similarity assessment with Bray-Curtis dissimilarity (function ‘metaMDS’). PERMANOVA tested group similarity significance. Alpha diversity was evaluated using Shannon index. Hierarchical clustering based on square-rooted Bray-Curtis distance was used to classify MAGs into clusters. Spearman’s rank correlation gauged relationships between decomposition rates and diversity/abundance across levels. Correlation tests examined associations between litter chemistry turnover and fungal/enzyme diversity/abundance. Multi-factor ANOVA were used to test the individual effects of plot, litter type, and incubation time on litter chemical properties, enzyme-coding gene abundances, and decomposition rates. Mann-Whitney U test assessed soil properties’ site and seasonal effects, and initial litter chemistry differences. Principal component analysis (PCA) extracted features from qualities, properties, and fungal composition. Significance was determined by adjusted *p*-values (Bonferroni method), considering <0.05 as significant.

## Results

### Decomposition rates and litter chemical dynamics

**Figure 1.**
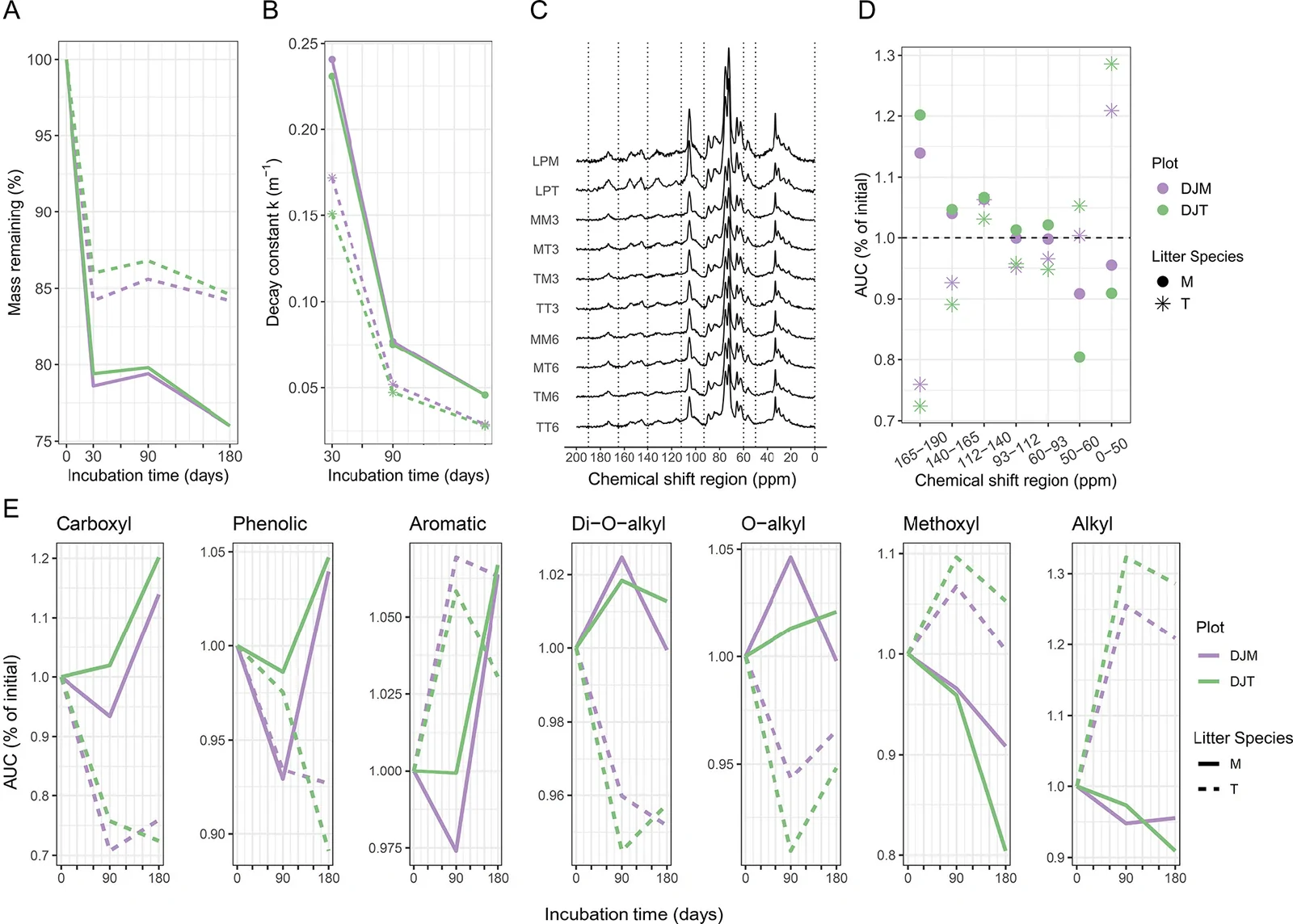
Litter decomposition. Mass remaining (**A**) and the decay constant *k* (**B**) over the course of 180 days of incubation. Panels A and B share a common legend. M denotes *P. morrisonicola* litter; T denotes *P. taiwanensis* litter. **C**^13^C NMR spectra. Chemical shifts were divided into seven regions representing different carbon types according to Berg and McClaugherty (2020). **D** Changing patterns of the seven carbon types after six months of decomposition. Chemical shift regions: 0–50, alkyl-C; 50–60, methoxyl-C; 60–93, O-alkyl-C; 93–112, di-O-alkyl-C; 112–140, aromatic-C; 140–165, phenolic-C; 165–190, carboxyl-C. Data were quantified by the area under the curve (AUC) calculated using the linear trapezoidal method. **E** Dynamics of different carbon types in the needle litter throughout the decomposition process. The same legend is used for panels A, B, and E.

Initial *P. morrisonicola* litter (M litter) had higher total N, but lower total S content, and a lower C:N ratio compared to *P. taiwanensis* litter (T litter). H contents were similar between the two species. Total C content and C:H ratios were relatively lower in the M litter, although these differences were not statistically significant (Mann-Whitney *U* test, *p* = 0.275 and *p* = 0.127, respectively) (Suppl. material 1: table S3; fig. S5). Water content in the litter decreased during decomposition; DJT-incubated litter experienced more substantial water loss compared to DJM-incubated litter, and the M litter retained more water after six months (Suppl. material 1: fig. S5B). Following 180 days of incubation, the decay constants displayed significant differences between litter species (Multi-factor ANOVA, *p* < 0.001), while plot-based differences were not significant (*p* > 0.05) (Suppl. material 1: table S4). Specifically, the M litter lost 24% of their mass at both plots, whereas the T litter lost only 16% and 15% of its mass in the DJM and DJT plots, respectively (Fig. 1A). T litter decomposed slightly faster in DJM compared to DJT plots. Decomposition rates declined over time, particularly during the first three months (Fig. 1B, Suppl. material 1: table S4). ^13^C NMR analysis revealed distinct dynamics of organic carbons during the 180-day incubation period (Fig. 1C). DJT-incubated litter displayed significant carbon type differences after six months (Fig. 1D). Over six months, Aromatic-C content increased for both species, while Alkyl-, Methoxyl-C, Phenolic-, and Carboxyl-C displayed opposing trends. *P. morrisonicola* showed an increase in Phenolic- and Carboxyl-C, coupled with a decrease in Alkyl- and Methoxyl-C, while *P. taiwanensis* showed the reverse. O-alkyl- and Di-O-alkyl-C content decreased in the T litter, showing varied patterns in the M litter (Fig. 1E, Suppl. material 1: fig. S6).

### Fungal composition and succession

**Figure 2.**
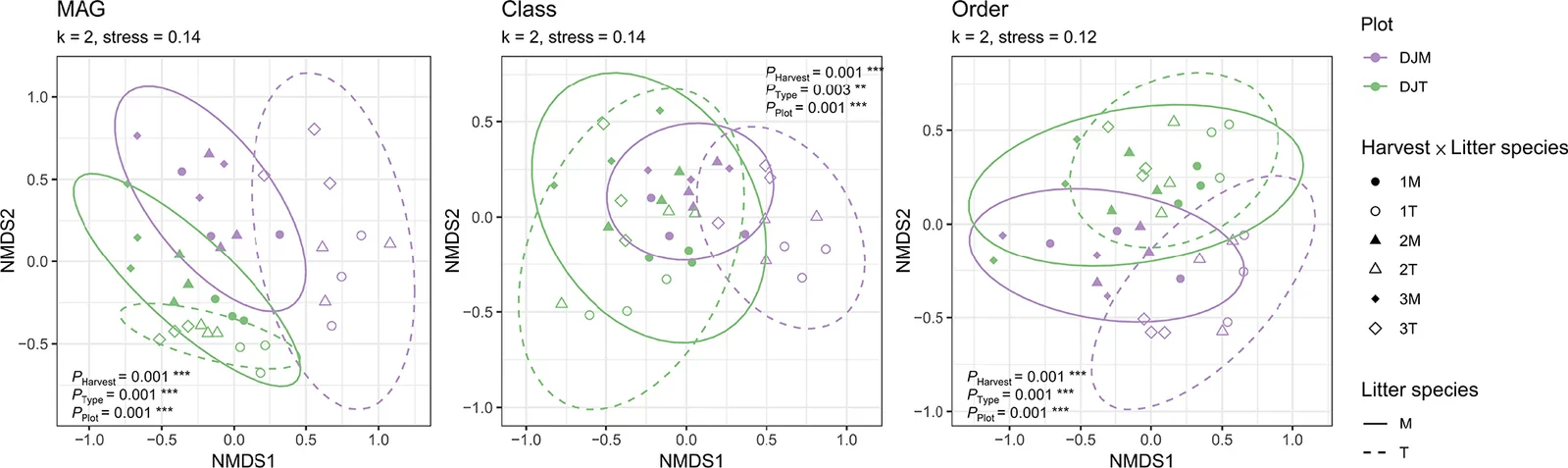
Non-metric multidimensional scaling (NMDS) of the fungal composition at distinct levels. The NMDS is based on Bray-Curtis dissimilarity matrices. Colors represent the different sampling plots, while shapes indicate the harvest times and the litter species.

From the metagenomic sequencing, we recovered 170 fungal MAGs, with BUSCO scores ranging from 5.5% to 98.5% (mean: 60.1%; median: 64.3%). Of these, 110 MAGs had BUSCO completeness >50%, including 26 MAGs with BUSCO completeness >90%. NMDS and PERMANOVA analyses revealed that the fungal community composition was significantly shaped by plot, litter species, and harvest time at the MAG, class, and order levels (Fig. 2; Suppl. material 1: table S5). During decomposition, overall alpha-diversity increased at the MAG level but decreased at class level (Suppl. material 1: fig. S7). Furthermore, the home-field M litter (DJM.M) exhibited the highest diversity across all sampling times and taxonomic scales.

**Figure 3.**
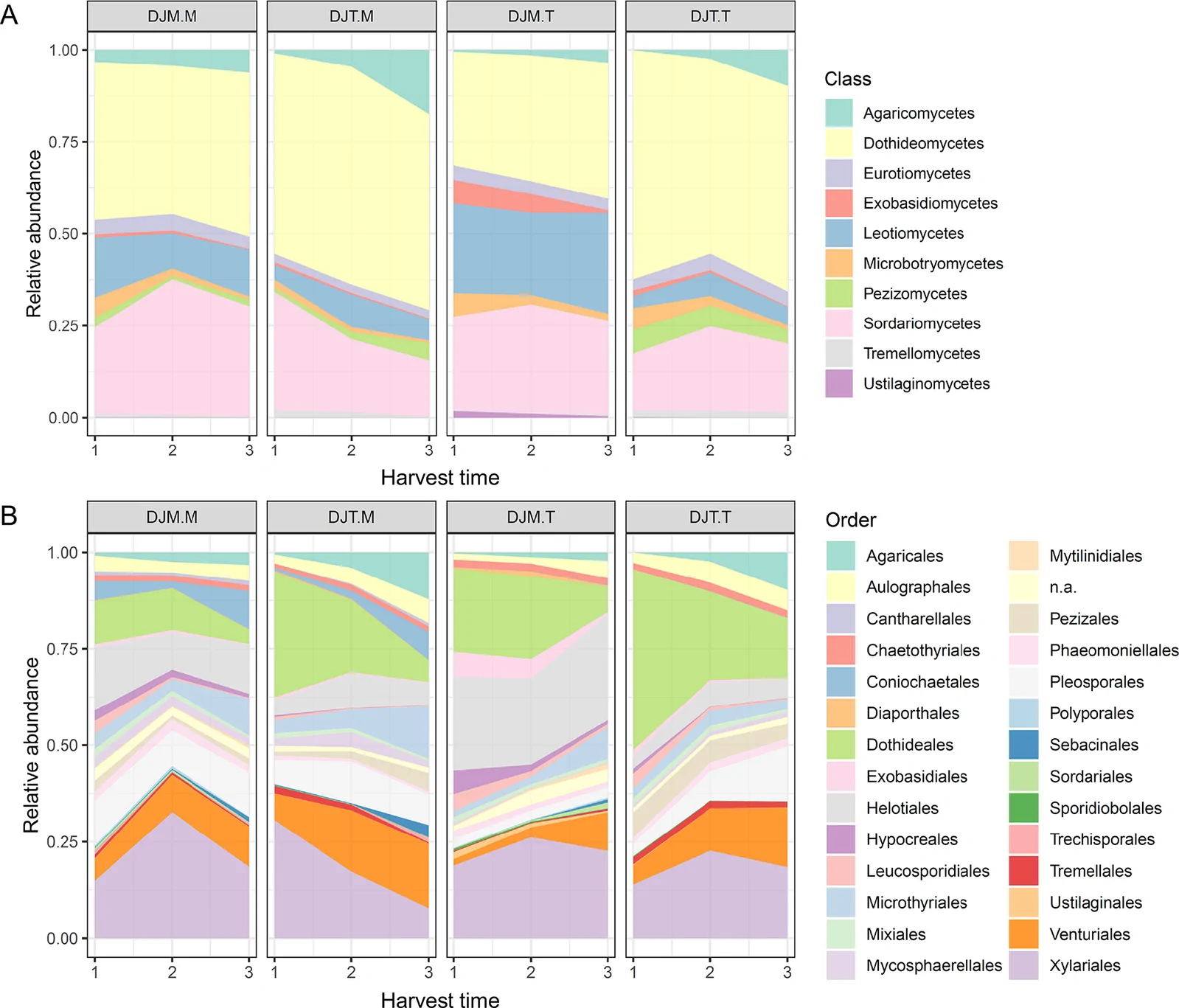
The succession of the distinct fungal taxonomy levels in four sample types throughout decomposition. DJM.M: Plot DJM with *P. morrisonicola* litter species; DJT.M: Plot DJT with *P. morrisonicola* litter species; DJM.T: Plot DJM with *P. taiwanensis* litter species; DJT.T: Plot DJT with *P. taiwanensis* litter species. (**A**) Class. (**B**) Order.

The most dominant fungal classes in the decomposing needles were *Dothideomycetes* and *Sordariomycetes*, which were especially abundant in the DJT-incubated litter samples (Fig. 3). In the DJM.T group, *Leotiomycetes* were comparably abundant to *Sordariomycetes* and *Dothideomycetes*, whereas in other samples, they were less prevalent. Additionally, DJM.T exhibited a notable presence of *Exobasidiomycetes*, which was rarely detected in the other samples (Fig. 3A). At the order level, *Dothideales*, *Xylariales*, *Venturiales*, and *Pleosporales* were the dominant orders in the DJT-incubated litter (Fig. 3B). At the DJM site, *Xylariales* dominated in the M litter, whereas the T litter exhibited similar proportions of *Helotiales* and *Xylariales*. The DJT-incubated litter contained higher proportions of *Agaricales*, *Dothideales*, and *Venturiales*; notably, the relative abundance of *Agaricales* increased during decomposition, while *Dothideales* decreased. Furthermore, *Dothideales* were more abundant in the T litter, while *Coniochaetales* and *Microthyriales* exhibited higher prevalence in the M litter. *Helotiales* were more abundant in the DJM-incubated litter than in the DJT-incubated litter (Fig. 3B).

**Figure 4.**
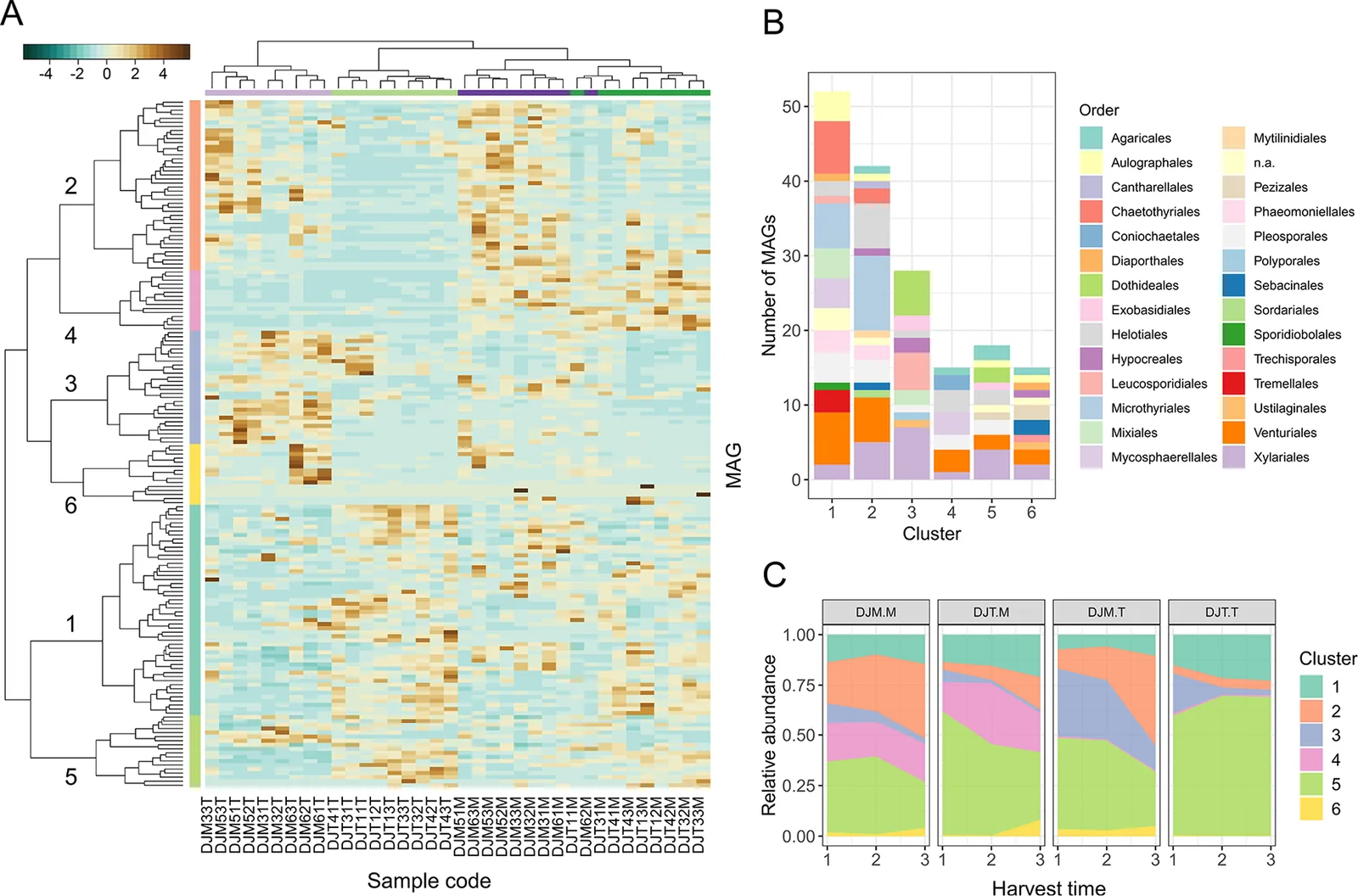
Distribution, taxonomic composition, and successional dynamics of MAG clusters during litter decomposition. **A** Heatmap of MAG distribution across all samples. Sample codes on the x-axis follow a specific structure: [Plot][Replicate][Harvest][Litter species]. Specifically, the first three letters indicate the plot (DJM or DJT); the first digit indicates the subplot replicate; the second digit indicates the harvest time; and the final letter denotes the litter species (M for *P. morrisonicola*, T for *P. taiwanensis*). For example, DJM33T denotes a *P. taiwanensis* litter sample from the DJM subplot replicate 3, collected at the third harvest. The row-side colors denote 6 groups of fungi identified by hierarchical clustering, and the column-side colors indicate plot DJM (purple), plot DJT (green), the M litter (dark color), and the T litter (light color); **B** Constitution of the 6 clusters at the order level. n.a.: not assigned; **C** Cluster succession in the four sample types throughout the decomposition process.

The fungal community was divided into six MAG clusters according to species distribution (Fig. 4A). *Xylariales* was ubiquitous, appearing in all clusters, while specialists such as *Tremellales* and *Sporidioborales* were specific to Cluster 1 (Fig. 4B). Cluster 5 maintained a high abundance across all sample types, reaching its maximum relative abundance in the DJT.T group. Cluster 3 dominated DJM.T but exhibited a continuous decline over the incubation period. The M litter uniquely hosted Cluster 4 fungi, rarely found in the T litter. Cluster 2 thrived in DJM-incubated litter, increasing over time, while DJT-incubated litter were characterized by a higher presence of Cluster 1 fungi. Cluster 6, although initially present at low levels in all samples, increased gradually throughout the decomposition process (Fig. 4C).

### Enzymatic profiles

After filtering out MAGs with BUSCO completeness below 30%, the remaining 139 MAGs (82% of the total) were utilized for downstream enzymatic analysis. Across all annotated fungal orders, *Sordariales* exhibited the highest gene copy numbers for cellulolytic and ligninolytic enzymes. Collectively, *Sordariales*, *Agaricales* and *Aulographales*, these three orders harbored the most abundant repertoires of lignocellulolytic enzymes (Suppl. material 1: fig. S8A, B). Notably, the order *Trechisporales* was specifically enriched in LiP enzymes (Suppl. material 1: fig. S8B).

**Figure 5.**
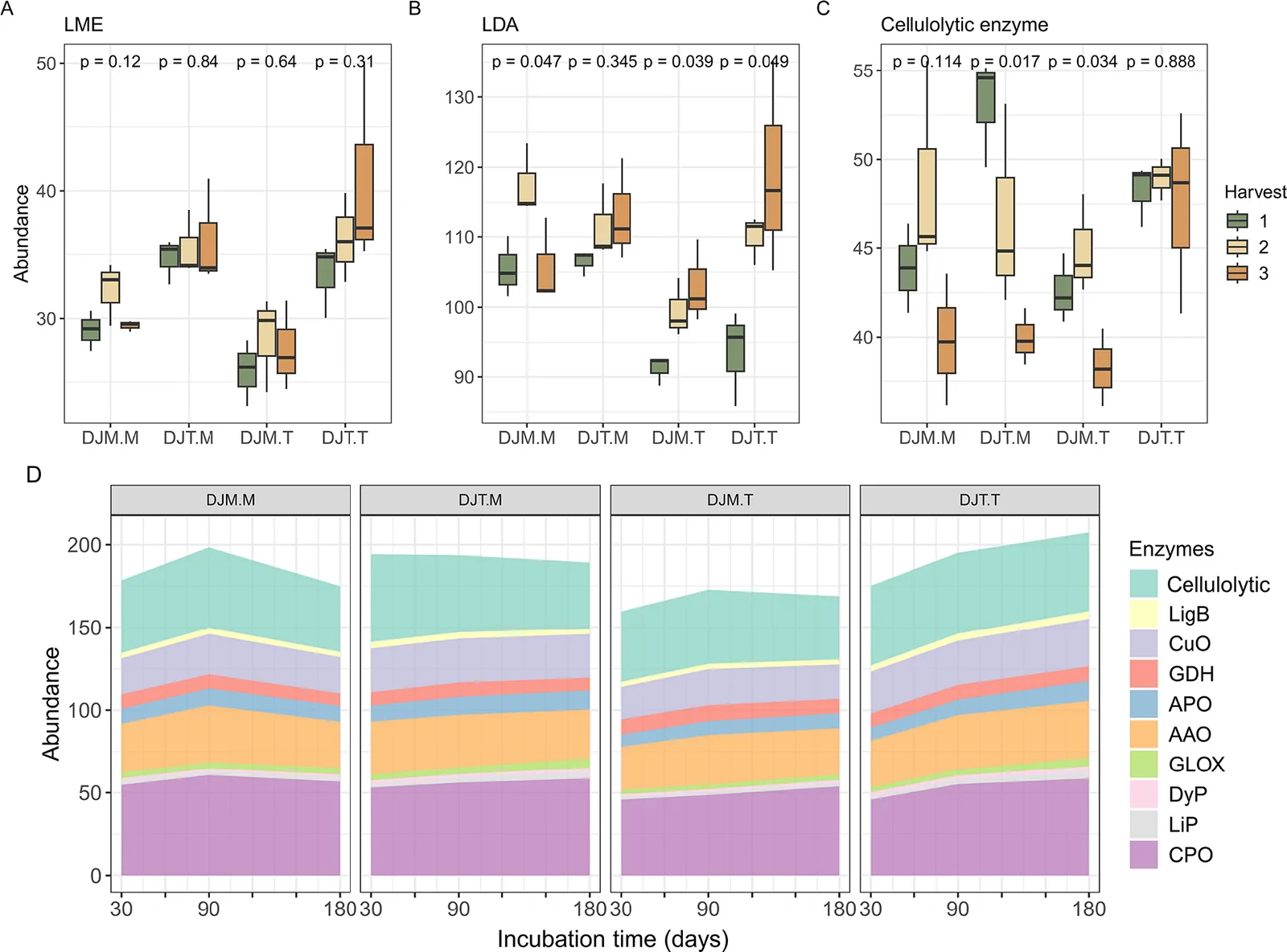
Enzyme abundance. Abundances of the ligninolytic enzymes categorized as LME (**A**), LDA (**B**), and cellulolytic (**C**) enzymes across sample types and harvests; **D** Progression of the enzyme composition throughout the decomposition process in each sample type. “Cellulolytic” refers to cellulose-degrading enzymes. The incubation times correspond to three harvest times in our experiment. Asterisks in panel **A–C** indicate statistically significant differences in abundance across harvest times based on one-way ANOVA (* *p* ≤ 0.05).

To simplify enzymatic dynamics, we categorized ligninolytic enzymes into two groups: lignin-modifying enzymes (LMEs: CuO, LiP, LigB, DyP) and lignin-degrading auxiliary enzymes (LDAs: GDH, APO, AAO, GLOX, CPO). Genes encoding LMEs exhibited higher abundance at the DJT site, while those encoding LDAs showed significant variations primarily across harvest times (Multi-factor ANOVA, *p* < 0.001) (Fig. 5; Suppl. material 1: table S6). Specifically, LDA gene abundance peaked at the third harvest for all sample types except DJM.M (Fig. 5A, B). Conversely, genes encoding cellulolytic enzymes decreased consistently throughout decomposition (Fig. 5C). Among the individual enzymes studied, CPO was the most prominent across all sample types, followed by AAO and CuO, whereas DyP, LiP, LigB, and GLOX were detected at lower levels. The collective gene abundance of cellulolytic enzymes, which encompasses five GHs, was comparable to that of CPO (Fig. 5D). Overall, over the six-month incubation, the relative abundance of both LMEs and LDAs increased as the decomposition progressed.

### Relationships between fungal communities and decomposition rates

**Figure 6.**
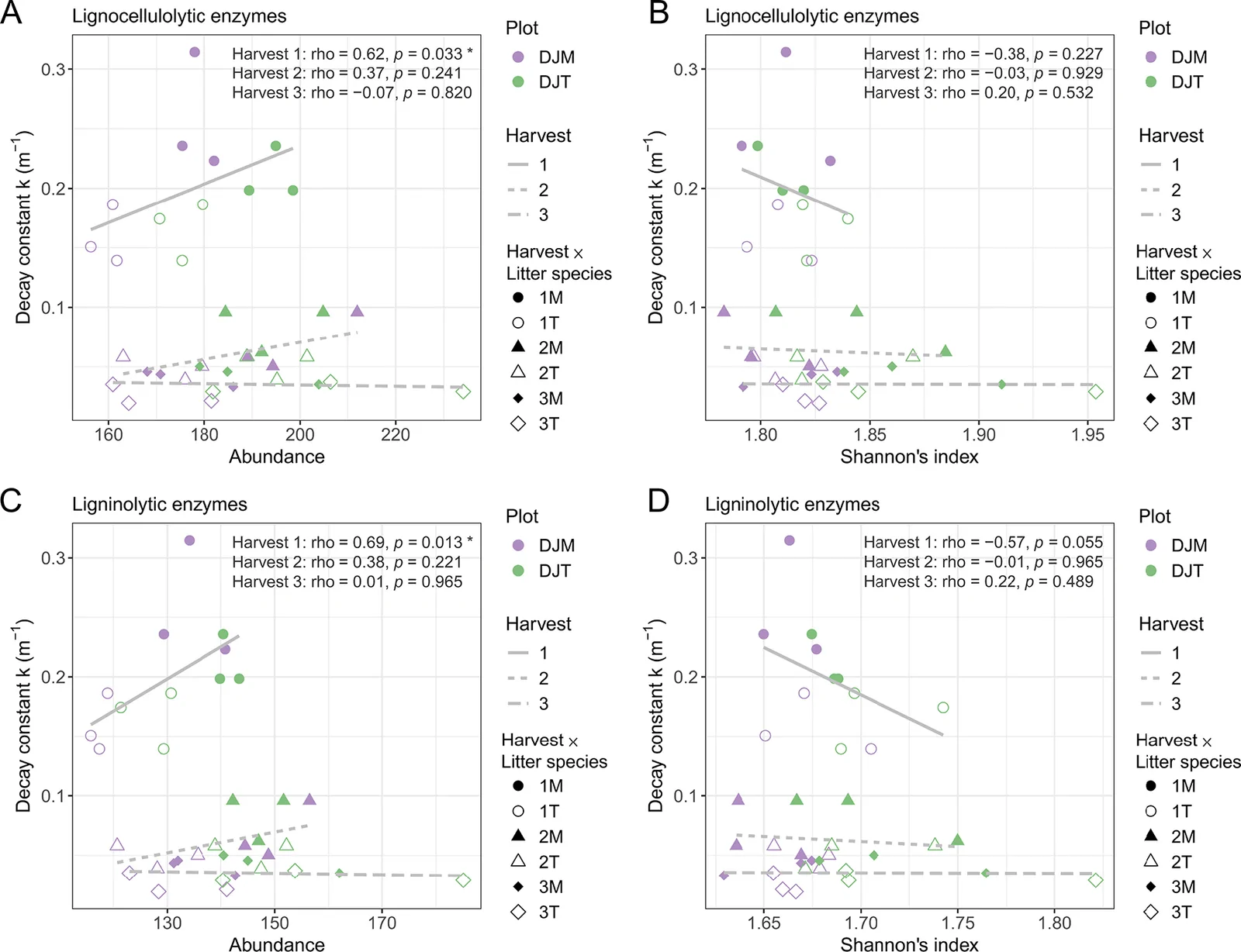
Correlation of decomposition rates with the lignocellulolytic and ligninolytic enzymes (LDE) abundance and diversity. Statistical significance and the strength of monotonic relationships were determined using Spearman’s rank correlation test (Suppl. material 1: table S7). Regression lines were fitted using the linear model for each harvest.

Overall, based on Spearman’s correlation analyses, total enzyme-coding gene abundance and diversity did not demonstrate broad significant correlations with decomposition rates (Fig. 6A, B; Suppl. material 1: table S7). However, a noteworthy positive correlation emerged between the abundance of genes encoding ligninolytic enzymes and decay constants, particularly in the first harvest (*ρ* = 0.69, *p* = 0.013) (Fig. 6C). Conversely, no significant correlation was identified for the diversities of genes encoding lignin-degrading enzymes (*ρ* = –0.01, *p* = 0.937) (Fig. 6D). Within the spectrum of nine lignin-degrading enzymes, intriguing patterns emerged. Specifically, APO correlated positively with decay constants in the second harvest (*ρ* = 0.65, *p* = 0.021). Furthermore, GLOX revealed positive correlations with decay constants in both the first (*ρ* = 0.77, *p* = 0.003) and second (*ρ* = 0.61, *p* = 0.036) harvests, while CPO exhibited a positive correlation solely in the first harvest (*ρ* = 0.79, *p* = 0.002). Notably, a distinct negative correlation was identified for GDH in the third harvest (*ρ* = –0.73, *p* = 0.007) (Suppl. material 1: fig. S9). To investigate the successional dynamic of the lignin modification, we filtered for MAGs possessing at least four of the five LMEs, specifically those encoding LiP and MnP (Suppl. material 1: fig. S10a). Of these, 74 MAGs carrying either MnP or LiP, along with at least two of the other three LMEs, were classified as potential ‘white-rot fungi’. A total of 20 MAGs carried both MnP and LiP, of which only four belonged to the phylum *Basidiomycota* (class *Agaricomycetes*) (Suppl. material 1: fig. S10b). The overall abundance of these potential white-rot fungi slightly increased as litter decomposition progressed. Notably, the different plots appeared to be dominated by distinct white-rot fungal communities. Additionally, MAG0200, assigned to the family *Dothioraceae*, appeared to play a key role in the early stages of decomposition across all plots. These shifts in enzyme abundance likely reflect contributions from fungal orders with distinct functional capacities. For instance, *Diaporthales*, *Mycosphaerellales*, and *Xylariales* exhibited strong genomic potential for both ligninolytic and cellulolytic decomposition. In contrast, *Agaricales* and *Aulographales* showed high ligninolytic potential, whereas *Sebacinales*, *Cantharellales*, and *Dothideales* were more proficient in cellulolytic degradation (Suppl. material 1: table S8).

## Discussion

### Litter decomposition dynamics driven by litter species traits and microclimate

In our 6-month litterbag experiment, we investigated the linkages among litter characteristics, fungal decomposer communities, and decomposition rates in two *Pinus* species with contrasting fire-adaptive strategies. We hypothesized that divergent litter traits between the fire-avoiding *P. morrisonicola* and fire-tolerant *P. taiwanensis* would drive distinct decomposition dynamics, mediated by specific fungal assemblages. Our findings supported this hypothesis: *P. morrisonicola* litter demonstrated accelerated mass loss over the 6-month incubation, driven by higher N content and a lower C:N ratio (Suppl. material 1: fig. S5). While Sanborn (2001) noted that broadleaf litter typically possesses lower C:N ratio than conifers, our results highlighted that significant functional variation exists even within conifer lineages. Moreover, our observations aligned with established paradigms that higher N availability and lower C:N ratios accelerate decomposition (Zhang et al. 2008; Grootemaat et al. 2015; Lin et al. 2019; Berg and McClaugherty 2020). During decomposition, the C:N ratio of both species decreased (Suppl. material 1: fig. S11), with *P. taiwanensis* exhibiting a more pronounced decline than *P. morrisonicola*. These stoichiometric shifts likely reflect underlying successional dynamics in the decomposing needle microbiome. A resource-rich environment (low initial C:N) fosters efficient decomposer taxa with robust lignocellulolytic capabilities, thereby promoting nutrient cycling and favoring biologically driven carbon turnover over fire-driven processes. Additionally, ^13^C-NMR spectroscopy revealed distinct decomposition trajectories between the two species (Fig. 1E). Specifically, the proportion of the 140–165 ppm chemical shift region, corresponding to phenolic carbons (Berg and McClaugherty 2020), decreased after six months exclusively in the *P. taiwanensis* litter. In contrast, the 112–140 ppm region, indicative of aromatic carbons, increased in both litter types, suggesting a relative accumulation of lignin as decomposition progressed. These diverging chemical trajectories directly influenced the functional output of the decomposer community. When evaluating the pooled data across both litter types (Fig. 6), the apparent relationships between the decay constant and lignocellulolytic enzyme gene abundance were likely modulated by these species-specific differences. At the first harvest, a significant positive correlation emerged between lignocellulolytic enzyme gene abundance and the decay constant. However, this overall slope was largely driven by litter identity rather than a continuous abundance-decay dynamic: *P. morrisonicola* litter inherently supported higher overall enzyme abundances and a faster initial decay trajectory compared to *P. taiwanensis* litter. Concurrently, fungal community diversity displayed only a slight, non-significant negative tendency with the decay constant during this early stage. As decomposition progressed to the second and third harvests, the enzyme-decay correlations weakened, and the regression slopes became progressively flatter. The distinct clustering of the two litter species along these regression axes underscores that litter identity acts as a primary environmental filter, dictating initial fungal recruitment and functional enzyme expression. Indeed, this strong filtering effect aligns with broader ecological studies across diverse ecosystems, which have consistently demonstrated that plant-derived conditions—such as specific nutrient stoichiometry and structural recalcitrance—fundamentally shape the assembly and enzymatic profiles of decomposer fungal communities (Gartner and Cardon 2004; Fernández-Alonso et al. 2022). Overall, these patterns indicate substantial interspecific differences in initial chemical properties and shape the subsequent fungal successional dynamics and enzymatic processing during early decomposition.

Beyond these litter-driven effects, forest stand identity also subtly modulated decomposition dynamics, offering intriguing insights into the home-field advantage (HFA) phenomenon (Ayres et al. 2009; Veen et al. 2019). Overall, litter incubated in the DJT stand exhibited a slightly slower mass reduction compared to the DJM stand (Fig. 1A). However, rather than strictly supporting a universal HFA hypothesis, our data suggest that strong HFA effects can be ruled out for these species during this early decomposition stage. Specifically, while the fire-avoiding *P. morrisonicola* litter displayed minor numerical variations in mass loss between its native and non-native stands, these differences were not statistically significant. Therefore, while stand identity might exert subtle influences, these effects were not pronounced enough to override the primary filtering effect of litter identity.

While litter identity served as the primary driver of decomposition—consistent with syntheses suggesting that stand characteristics generally exert a secondary influence on local variations (Wang et al. 2015)—stand identity remains a non-negligible factor. This hierarchy of controls was corroborated by our ^13^C-NMR data, which indicated that litter type primarily dictated chemical shifts during early decomposition, whereas stand identity drove intraspecific variation across sites. Nonetheless, as noted by De Santo et al. (1993), microclimatic conditions such as moisture can occasionally supersede litter quality in driving decomposition patterns. In the DJM forest, higher humidity and canopy coverage likely enhanced overall fungal activity (Pressler et al. 2019; Nelson et al. 2022). In addition to these abiotic factors, the greater structural complexity and fungal diversity of the mixed-forest floor probably facilitated needle decomposition, reinforcing that stand identity is an integral element shaping local biogeochemical cycles.

### Taxonomic succession of lignocellulolytic fungi

The distinct chemical trajectories and microclimatic influences discussed above are fundamentally driven by the successional dynamics of the lignocellulolytic fungal communities. Litter decomposition typically proceeds through an initial cellulose-degradation phase followed by a late-stage lignocellulose-degradation phase. During the early phase, *Ascomycota*, particularly *Dothideomycetes* and *Leotiomycetes*, are widely reported to dominate (Osono 2006; Lindahl and Boberg 2008; Liers et al. 2011; Miao et al. 2022). Our taxonomic profiling aligned with this established trajectory (Fig. 3A). Across both pine litter types, *Ascomycota* (with *Dothideomycetes* as the most predominant class) dominated during the first six months. Consistent with the findings of Wang et al. (2023) and Hu et al. (2025), we observed a relative decrease in *Dothideomycetes* paired with an increase in *Sordariomycetes* as decomposition progressed. Subsequently, *Basidiomycota* became enriched, reflecting the functional shift toward late-stage decomposition. To validate the specific roles of these taxa, our metagenomic profiling revealed that MAGs strongly enriched in *P. morrisonicola* litter (Cluster 4)—which included members of *Agaricales* and *Xylariales*—possessed a broad repertoire of lignocellulolytic genes (Suppl. material 1: table S8). While culture-based studies have long validated the strong cellulolytic abilities of *Xylariaceae* (Rogers 1979; Nilsson et al. 1989), our genomic data further demonstrated that *Xylariales* ranked prominently in both cellulolytic (sixth) and ligninolytic (seventh) gene counts, indicating a versatile and notable role in overall litter breakdown (Suppl. material 1: table S8). The ligninolytic capacity of this fungal group is also supported by previous studies of *Xylariaceae* (Urairuj et al. 2003). Furthermore, the relative abundance of *Agaricales* surged at the six-month mark, especially in litter incubated within the *P. taiwanensis* forest. Recognized as potent wood decomposers (Floudas et al. 2020), *Agaricales* harbored the highest average number of ligninolytic enzyme genes, despite ranking lower in the cellulolytic potential (ninth), directly confirming their specialization in late-stage lignin degradation.

As easily decomposable substrates (e.g., cellulose and hemicellulose) become scarce, fungal communities must pivot their focus toward lignin degradation. Because lignin depolymerization necessitates a complex suite of specialized enzymes, we observed a heightened diversity and abundance of genes encoding these enzymes during these later stages (Fig. 5). This trend was more pronounced in the *P. taiwanensis* forest (Suppl. material 1: fig. S12), implying a direct adaptation of the resident microbiomes to the more recalcitrant nature of *P. taiwanensis* litter. In contrast, the litter incubated in the *P. morrisonicola* forest exhibited comparatively lower overall abundances of these enzyme-coding genes. However, such lower lignin degradation potential was likely compensated by the stand’s elevated humidity and denser canopy coverage, which sustain robust fungal activity (Fioretto et al. 2005; Krishna and Mohan 2017). This compensatory microclimate effect explains our observation that *P. taiwanensis* litter actually decomposed faster when reciprocally incubated in the *P. morrisonicola* forest than in its native site. Ultimately, these intricate microbiome-environment interactions align with the divergent fire-adaptive strategy of the two species. The fire-avoiding *P. morrisonicola* represent a humid, high-turnover environment with inherently lower litterfall accumulation (Suppl. material 1: fig. S13), which leads to lesser fuel loads and thereby lower forest susceptibility to fire. Conversely, the fire-tolerant *P. taiwanensis* produces recalcitrant litter that specifically selects for a diverse, lignin-specialized fungal community to drive its distinct decomposition dynamics.

### From latent saprotrophs to lignin specialists: genomic resolution of functional dynamics

A striking feature of our metagenomic dataset was the overwhelming dominance of *Ascomycota*, which accounted for 81% of the recovered fungi and served as the primary source of genes encoding lignocellulose-degrading enzymes. While soft-rot ascomycetes generally possess weaker direct decomposition capabilities compared to white- and brown-rot basidiomycetes, their production of organic acids can significantly stimulate the decomposition process (Lundell et al. 2014). Particularly in the DJM forest, *P. taiwanensis* litter was heavily colonized by *Leotiomycetes*. Recognized as early-stage decomposers of biopolymer decomposers like cellulose (Osono 2006; Schneider et al. 2012), many ascomycetes are also frequently identified as leaf endophytes. Given that our experimental needles were initially sterilized via heating to eliminate background microbiomes, the heavy colonization by these taxa indicates a strong external origin. We propose that the continuous input of newly fallen leaves from the canopy served as a primary source of fungal inoculum. Leaf endophytes are known to exhibit outstanding stress tolerance in the harsh phyllosphere (Voříšková and Baldrian 2013; Baskaran et al. 2019; Zhan et al. 2021) and often adopt a “latent saprotrophic” lifestyle with leaf senescence (Rajala et al. 2014). Consequently, dormant saprobes from fresh surrounding litterfall likely rapidly colonized the experimental litterbags, acting as a reservoir of early-stage decomposing microorganisms. In addition, the ability of these ascomycetes to synthesize nitrogen-rich melanins may drive the N immobilization observed during early decomposition (Baskaran et al. 2019).

As the availability of labile substrates shifted over time, we observed distinct successional dynamics in the functional gene profiles. Overall, the abundance of genes encoding cellulolytic enzymes decreased over the 6-month period (Fig. 5C), suggesting the depletion of cellulose. However, species-specific enzymatic requirements emerged: the *P. taiwanensis* litter exhibited a significant increase in genes encoding lignin-degrading enzymes, whereas the *P. morrisonicola* litter showed a notable reduction in cellulolytic gene abundances. This functional divergence indirectly reflects the successional transition among different wood-decaying guilds, particularly the increasing involvement of white-rot and brown-rot fungi. *Agaricomycetes* (mushroom-forming fungi) are well-documented for their wood-decaying proficiencies (Nagy et al. 2016; Yuan et al. 2023). Within our dataset, *Trechisporales* (mostly Cluster 6), a potential white-rot lineage encoding manganese peroxidase (MnP), harbored the highest number of lignin peroxidase (LiP) genes alongside other lignin-modifying enzymes (LMEs). The relative abundance of Cluster 6 steadily increased throughout decomposition (except in the DJT.T samples), underscoring its pivotal role in breaking down recalcitrant compounds. Because cellulose degradation within a complex lignocellulose matrix relies on synergistic action of both cellulolytic and ligninolytic enzymes, enzymatic diversity becomes increasingly vital as decomposition proceeds (Janusz et al. 2017). For instance, lignin-modifying GLOX and auxiliary APO and CPO likely contributed to the discrepancies observed in early decomposition rates. Interestingly, we noted a negative correlation between overall decay constants and the abundance of glucose dehydrogenase (GDH) genes at the 6-month mark. This may be attributed to the fact that GDH utilizes substances like phenoxy radicals and quinones—rather than oxygen—as external electron acceptors during specific degradation pathways (Janusz et al. 2017).

Ultimately, the temporal surge in the abundance of genes encoding ligninolytic enzymes, coupled with the decline in those encoding cellulolytic enzymes, precisely mirrors established empirical models of litter decomposition. This suggests that genome-resolved metagenomic sequencing can accurately approximate real-world fungal and enzymatic activities in forest ecosystems. Although our analyzed dataset was constrained to a 6-month incubation period—as the experiment was prematurely terminated by a severe wildfire in the ninth month—it clearly delineates profound, species-specific decomposition trajectories between *P. taiwanensis* and *P. morrisonicola*. These distinctions manifest not only in mass loss rates and litter chemistry but also in the underlying successional dynamics and functional enzyme profiles of their adapted microbiomes. In a broader ecological context, these structure-function relationships are intricately linked to the historical fire dynamics of their native ecosystems. In fire-tolerant ecosystems, frequent fire recurrences drive periodic fungal turnover, which likely disrupts long-term fungal successions and limits the establishment of late-stage ligninolytic specialists natively. Conversely, the stable, low-disturbance environments of fire-avoiding species provide the necessary temporal continuity for complex fungal networks to develop, allowing specialized fungal communities to effectively partition late-stage decomposition tasks. Therefore, a plant’s divergent fire-adaptive strategy not only governs its foliar traits and flammability but also fundamentally shapes the successional trajectories and enzymatic potential of its decomposer mycobiome.

## Conclusion

Our study underscores the critical role of litter decomposition in shaping fuel bed formation and, consequently, regulating fire regimes. By comparing two pine species with contrasting eco-evolutionary strategies—the fire-tolerant *P. taiwanensis* and the fire-avoiding *P. morrisonicola—*we confirmed that the latter exhibits accelerated decomposition rates. This disparity is fundamentally driven by the synergistic interaction between litter chemistry and mycobiome assembly. While the local stand environment dictates the broader pool of fungi, the specific chemical traits of the litter act as a strong environmental filter, recruiting distinct assemblages of lignocellulolytic fungi. Specifically, the expedited decomposition observed in the fire-avoiding pine is attributed to its inherently lower litter recalcitrance combined with its ability to rapidly recruit highly efficient early-stage decomposers. Furthermore, our genome-resolved metagenomic profiling demonstrates that the abundance of genes encoding specific lignocellulolytic enzymes accurately reflects these distinct decomposition dynamics. Finally, by integrating taxonomic composition with functional gene profiles, this study provides a comprehensive mechanistic understanding of how plant-fungal interactions govern biogeochemical cycling and ecosystem-level fire strategies.

## Additional information

### Artificial Intelligence (AI) use

The authors accept full responsibility for the content of the manuscript, including the disclosure of any use of AI.

Regarding the use of AI in the preparation of this manuscript, the authors declare the following: Gemini, GPT

Used for: Language, style and writing

We used AI tools to improve the writing of this manuscript.

### Adherence to national and international regulations

All the fungal strains used in this study have been legally obtained, respecting the Convention on Biological Diversity (Rio Convention).

### Author ORCIDs

Y.-H. Fang https://orcid.org/0009-0008-2400-4600

H.-N. Liu https://orcid.org/0009-0004-2961-7677

C.-A. Chiu https://orcid.org/0000-0002-6134-0530

P.-N. Chiang https://orcid.org/0000-0003-4269-3186

C.-L. Huang https://orcid.org/0000-0002-1059-432X

### Data availability

Shotgun metagenomic sequences are available at NCBI under SRA project PRJNA1465023.

## Supplementary materials

Supplementary material 1 Additional tables Data type xlsx Explanation note **table S1**. Taxonomic assignment matrix of fungal MAGs. **table S2**. MAG with a BUSCO completeness score greater than 30%. **table S3**. Initial litter chemistry (mean ± sd) with Mann-Whitney U tests for differences between litter species. **table S4**. ANOVA on decay constants to test the effects from plot, litter species, harvests, and their interactions. **table S5**. PERMANOVA on fungal compositions at MAG, class, and order levels. **table S6**. ANOVA on abundance of cellulolytic and lignin-degrading enzymes. **table S7**. Spearman’s correlation between decay constant *k* and the abundances and diversities of enzymes, in respect of three harvests. **table S8**. Top 27 orders with lignocellulolytic capacity. https://binary.pensoft.net/file/1802694This dataset is made available under the Open Database License (http://opendatacommons.org/licenses/odbl/1.0/). The Open Database License (ODbL) is a license agreement intended to allow users to freely share, modify, and use this Dataset while maintaining this same freedom for others, provided that the original source and author(s) are credited. Yan-Han Fang, Hsin-Ni Liu, Yen-Ju Chen, Tsai-Wen Hsu, Ching-An Chiu, Po-Neng Chiang, Chao-Li Huang

Supplementary material 2 Additional figures Data type docx Explanation note **fig. S1**. Records of temperature in experimental plots. **fig. S2**. Records of relative humidity in experimental plots. **fig. S3**. Experimental design, timeline, and laboratory workflow of the reciprocal litterbag study. **fig. S4**. Phylogeny of genes with LMEs. **fig. S5**. Physical properties of litter. **fig. S6**. Alkyl-C-to-O-Alkyl-C ratio of litter across distinct harvests. **fig. S7**. Alpha-diversity at the (A) MAG, (B) class, and (C) order levels. **fig. S8**. Enzyme copies in fungi. **fig. S9**. Correlation between decomposition rates and the abundances of nine target lignin-degrading enzymes for three harvests. **fig. S10**. MAGs with lignin-modifying enzymes. **fig. S11**. C/N and C/H ratio of litter across distinct harvests. **fig. S12**. Alpha-diversity of the lignin-degrading enzymes along the litter decomposition. **fig. S13**. Monthly litterfall accounting (A) total mass and (B) leaf mass. https://binary.pensoft.net/file/1802695This dataset is made available under the Open Database License (http://opendatacommons.org/licenses/odbl/1.0/). The Open Database License (ODbL) is a license agreement intended to allow users to freely share, modify, and use this Dataset while maintaining this same freedom for others, provided that the original source and author(s) are credited. Yan-Han Fang, Hsin-Ni Liu, Yen-Ju Chen, Tsai-Wen Hsu, Ching-An Chiu, Po-Neng Chiang, Chao-Li Huang
