# Supplementary material for: Metagenomic profiling reveals that divergent fire-adaptive strategies drive distinct fungal succession in pine litter decomposition
Source: IMA Fungus. 2026 Oct 6;17:e201414. doi: 10.3897/imafungus.17.201414 (PMC13662637; doi:10.3897/imafungus.17.201414)
Supplement: Supplementary file 1 [file imafungus-17-e201414-s002.docx]

**Fig. S1** **Records of temperature in experimental plots.** Data from DJT was missing from Dec 5, 2020 to Jan 18, 2021.

**Fig. S2** **Records of relative humidity in experimental plots.** Data from DJT was missing from Dec 5, 2020 to Jan 18, 2021.

**Fig. S3** **Experimental design, timeline, and laboratory workflow of the reciprocal litterbag study.** Two experimental forest stands—mixed forest (DJM) and pine forest (DJT)—each feature five replicate subplots containing six *P. morrisonicola* (M) and six *P. taiwanensis* (T) litterbags (canopy photographs shown at the top left of each panel). Temperature and humidity were recorded continuously at each site via a data logger. The two-year decomposition timeline includes sampling at months 1, 3, 6, 9, 12, 18, and 24, with an unexpected forest fire disturbance occurring at month 9. Post-collection laboratory workflows include storing samples at -80°C for microbial DNA analysis or oven-drying them for downstream biomass and chemical analysis.

**Fig. S4 Phylogeny of genes with LMEs.** The dots denote the LME carriers: MnP (cyan-blue) and LiP_VP (salmon).

**Fig. S5 Physical properties of litter.** (A) Total C, H, N and S contents detected in the initial litters, and the calculated C:N and C:H ratios. (B) Litter water contents after 30, 90 and 180 days of decomposition (1st, 2nd and 3rd harvests, respectively).

**Fig. S6 Alkyl-C-to-O-Alkyl-C ratio of litter across distinct harvests.**

**Fig. S7 Alpha-diversity at the (A) MAG, (B) class, and (C) order levels.** Colors and line types denote plots and litter species, respectively.

**Fig. S8** **Enzyme copies in fungi.** **A** Distribution of fungal orders across 6 clusters, and the mean gene copies of cellulolytic and ligninolytic enzymes of each order. n.a.: not assigned; **B** Heatmap of enzyme gene copies across the fungal orders with order abundances fitted below. The column-side color denotes the classes. n.a.: not assigned.

**Fig. S9 Correlation between decomposition rates and the abundances of nine target lignin-degrading enzymes for three harvests.** Statistical significance and the strength of monotonic relationships were determined using Spearman's rank correlation test (Suppl. material 1: table S7). Regression lines were fitted using the linear model for each harvest.

**Fig. S10 MAGs with lignin-modifying enzymes.** (A) Number of LME carriers; (B) Successional dynamics of putative white-rot fungi (MnP possessors). The left annotation indicated the MAGs with different LME carriers, while the right annotation indicated the phylum of these selected MAGs. The top annotation labeled the different sampling plots and litter types.

**Fig. S11 C/N and C/H ratio of litter across distinct harvests.**

**Fig. S12 Alpha-diversity of the lignin-degrading enzymes along the litter decomposition.**

**Fig. S13** **Monthly litterfall accounting (A) total mass and (B) leaf mass.**
